# The Impact of Signals Transmission on Patients’ Choice through E-Consultation Websites: An Econometric Analysis of Secondary Datasets

**DOI:** 10.3390/ijerph18105192

**Published:** 2021-05-13

**Authors:** Adnan Muhammad Shah, Rizwan Ali Naqvi, Ok-Ran Jeong

**Affiliations:** 1 Department of Information Technology, University of Sialkot, Sialkot 51310, Pakistan; 2 Department of Unmanned Vehicle Engineering, Sejong University, Seoul 05006, Korea; rizwanali@sejong.ac.kr; 3 School of Computing, Gachon University, Seongnam 13120, Korea

**Keywords:** e-consultation, COVID-19, signaling theory, patients’ choice, panel data analysis

## Abstract

(1) Background: The COVID-19 pandemic has dramatically and rapidly changed the overall picture of healthcare in the way how doctors care for their patients. Due to the significant strain on hospitals and medical facilities, the popularity of web-based medical consultation has drawn the focus of researchers during the deadly coronavirus disease (COVID-19) in the United States. Healthcare organizations are now reacting to COVID-19 by rapidly adopting new tools and innovations such as e-consultation platforms, which refer to the delivery of healthcare services digitally or remotely using digital technology to treat patients. However, patients’ utilization of different signal transmission mechanisms to seek medical advice through e-consultation websites has not been discussed during the pandemic. This paper examines the impact of different online signals (online reputation and online effort), offline signals (offline reputation) and disease risk on patients’ physician selection choice for e-consultation during the COVID-19 crisis. (2) Methods: Drawing on signaling theory, a theoretical model was developed to explore the antecedents of patients’ e-consultation choice toward a specific physician. The model was tested using 3-times panel data sets, covering 4231 physicians on Healthgrades and Vitals websites during the pandemic months of January, March and May 2020. (3) Results: The findings suggested that online reputation, online effort and disease risk were positively related to patients’ online physician selection. The disease risk has also affected patients’ e-consultation choice. A high-risk disease positively moderates the relationship between online reputation and patients’ e-consultation choice, which means market signals (online reputation) are more influential than seller signals (offline reputation and online effort). Hence, market signals strengthened the effect in the case of high-risk disease. (4) Conclusions: The findings of this study provide practical suggestions for physicians, platform developers and policymakers in online environments to improve their service quality during the crisis. This article offers a practical guide on using emerging technology to provide virtual care during the pandemic. This study also provides implications for government officials and doctors on the potentials of consolidating virtual care solutions in the near future in order to contribute to the integration of emerging technology into healthcare.

## 1. Introduction

In late December 2019, unknown pandemic cases emerged in Wuhan, China, and have become a big public health problem not only for China but countries all over the world [1]. The pandemic was declared coronavirus pathogen (COVID-19) by the World Health Organization (WHO) in mid-February [2]. After the spread of the pandemic to dozens of countries, including the United States (U.S.) [3], where 2,537,636 confirmed cases with 126,203 mortalities were reported at the time of writing (i.e., 27 June 2020) by the Centre for Diseases Control and Prevention (CDC) [4]. Obviously, the shortage and overloading of healthcare facilities have been the main concern during the outbreak [5]. In addition, delivering timely and efficient healthcare information and services seemed daunting during this pandemic because of insufficient protective gear, lockdown and the danger of spreading the infection to patients and doctors [6]. In order to reduce and control the spread of the pandemic, hospitals can improve their productivity of medical information systems by replacing traditional information systems with virtual platforms [7]. Virtual platforms such as e-consultation websites offer a novel approach to resolving the difficult situation between doctors and patients. These platforms also provide patients with essential details, help them find physicians with a specific style, assist them in their decision-making process, and make them better prepared for their future online consultation [8].

E-consultation, a revolutionary way to meet rising medical demand, helps users resolve barriers of space and time to provide more options for selecting doctors around the world, and it is becoming more widely used these days [9]. Many countries have implemented online health consultation services in order to offer better treatment to patients. One of the most popular platforms to search health-related information is e-consultation websites [10]. A web-based consultation can be useful to supplement conventional healthcare services and traditional doctor-patient relationships [11]. Scholars have indicated that between 25% and 70% of all patients seeking treatment do not want a face-to-face visit with a healthcare provider due to the high demand for online consulting services [12]. One of the major benefits of this modern form of e-consultation service is the minimization of both waiting time and travel costs [13]. These virtual care services offer a wide range of non-dispensing services, allowing doctors to provide high-quality medical care during the COVID-19 pandemic. This most recent type of virtual care can be incorporated into the healthcare system as a way to improve healthcare delivery quality [14]. It encourages social distancing and aids hospitals and clinics in coping with long wait times and the possibility of disease progression. By reducing physical visits and minimal face-to-face interaction among doctors and patients, virtual care systems will support reducing virus transmission and protecting medical professionals from infection [5]. Currently, COVID-19 is causing several medical facilities to cancel or postpone in-person outpatient medical appointments [15]. These e-consultation websites are also important in minimizing medical costs, enhancing clinical quality and efficacy, increasing the equity of medical services and meeting patient satisfaction. In order to make these e-consultation websites more effective for health consumers, it is necessary to adjust the contents of e-consultation websites to match the specific information needs according to the requirements of patients [16]. Due to the above reasons, e-consultation websites are the precious gold mines for patients regarding different aspects of healthcare during the COVID-19 pandemic [17].

Our research context is worth remembering as the healthcare industry is unique, since information asymmetry is fiercer in the traditional health care environment [18]. In a broader domain, information asymmetry represents a state in which few people have more knowledge than others [19]. Information asymmetry is one of the principal causes of disputes in doctor-patient relationships within conventional medical systems [20]. For instance, a competent doctor tends to convey quality information about the clinical service to patients by providing the best medical facilities. From the perspective of signaling theory in healthcare, (1) information asymmetry between a signaler and receiver occurs when physicians know more than patients, which leads to fraudulent activities by physicians to cheat their patients. (2) A potential conflict of interest between the signaler (physicians) and receiver (patients) may occur when physicians cannot serve the best interest of patients and take advantage of information asymmetry. For example, physicians who run their own clinics or treatment centers may prefer to refer patients to their facilities for needless care and gain financial benefits from doing so [20]. Without satisfying either of these two conditions, there is no need for signals because the problem lies in communication between both parties. When both these conditions are satisfied simultaneously, signals are generated by signalers toward receivers to prove their quality of medical service. A highly competent physician can communicate credible and quality information to their patients [21]. The worth of these informational signals depends on whether highly competent physicians can isolate themselves from less competent physicians. On e-consultation website, patients (receiver) who are intending to consult doctors, seeking different signals from doctors (signalers) about their health condition [22]. Hence, it is important in the virtual platforms that both doctors and patients should transmit different signals to minimize the information asymmetry by fostering the doctor-patient relationship strength.

In our online consultation context, the role of the signaling mechanism becomes important because doctors use intelligent signaling mechanisms not only to communicate socially with patients but also to show their expertise. This strategy is in line with the signaling literature in game theory [19]: by sending signals, one party can credibly pass on some private information about itself to another party. E-consultation websites display both online and offline signals that users can observe to understand the physician quality [23]. Online signals are obtained from virtual consultation-based platforms, including online reputation (market signals) and online effort (seller signals). In contrast, offline signals are derived from the offline setting, such as offline reputation (seller signals). The seller signal is the conventional signal clearly described in the previous signaling studies that the seller deliberately transmits to the buyer [23]. On the other hand, the market signal is the indicator or the information exchanged unconsciously or unknowingly between different players in the market [16]. The market signal as online word of mouth (WOM) is information regarding different products and services created by buyers based on the consumption experience. An example of the market signal is the information flow motivating the herding attitude of stakeholders [24].

Patients and physicians display different signals on e-consultation websites, affecting patients’ cognitive abilities to choose a good doctor [16]. People obtain information regarding the quality of healthcare services rendered by physicians through different signaling mechanism [25], such as the process that the signaler (i.e., the physician) sends measurable signals (i.e., offline reputation as physician title, education, experience and online effort) to the recipient (i.e., patients) to communicate details about the non-observable qualities (i.e., competency) in order to minimize information asymmetry [21,26]. Earlier research indicated that receivers are sovereign decision-makers [27]. Therefore, these virtual platforms permit patients to express their satisfaction and dissatisfaction toward a signaler by writing positive or negative reviews [28]. The patients’ feedback is reflected as the affective reactions of peers toward their experience with the quality of service that s/he gets from that doctor [29]. For these reasons, it is also important to consider the role of online signals (i.e., online reputation and online effort) and offline signals (offline reputation) generated by markets and sellers on patients’ choice of a physician on e-consultation platforms. Based on the assessment of these two measures, patients make more rational decisions while selecting their doctor online for e-consultation.

Compared with face-to-face consultation, e-consultation websites could provide patients with timelier and more convenient information by displaying different signals that could be helpful in patients’ physician selection choice for online consultation [30,31]. These signals include physician online reputation, offline reputation and online efforts. For example, Shah, et al. [32] found that online reputation in the form of patient-generated signals positively impacts the patients’ online decision-making process. A study by Liu, et al. [33] do demonstrate that individual physician offline and online reputations on e-consultation websites were positively associated with the patients’ choice to book appointments online with that particular physician. Hong, et al. [26] indicated that online reputation and knowledge contribution as market signals and seller signals significantly positively influence patients’ online choice to pay higher prices to physicians. In another study, the impact of seller signals (self-representation) and market signals (reputation) in e-consultation market is found significantly positive on physician volume of online bookings [16]. Zhang, et al. [34] found that the higher a family doctor’s online reputation and knowledge contributions on virtual platforms, the greater that his/her private benefits. Khurana, Qiu and Kumar [20] reported that doctors’ online responses as knowledge contribution on online healthcare portal could be regarded as a significant signal of clinical service quality. Furthermore, Wu and Lu [35] indicated that physician online reputation virtual platforms reflect as a signal of quality. Deng, et al. [36] revealed that patients’ choice is significantly affected by the physician reputation and effort online. Greenwood, et al. [37] showed that physician offline reputation in the form of high-level expertise significantly influences physician behavior to adopt new information in an online environment. Wu and Deng [38] revealed the positive correlation between physician capital as offline reputation and patients’ choice of online physician selection. In addition, physician reputation and benevolence as an effort online were found positively related to the patient assessment of physician performance [39]. Moreover, Li, Zhang, Ma and Liu [10] also indicated physician online reputation and self-representation as information signals in the e-consultation market. Several other studies also indicated that online reputation in the form of online physician reviews affects patients’ choice to choose the best doctor online [40,41,42,43]. Keeping in view the notion behind signaling theory, signalers (physicians) transmit signals to receivers (patients). Patients can then evaluate these signals to choose an expert healthcare provider who can provide them quality health services.

Based on the earlier discussion, the following research gaps are identified in the previous literature regarding the signaling applied in the online healthcare market. First, as mentioned above, many studies focused on the effects of different signals transmission on patients’ choice for online consultation in the pre-COVID-19 scenario. Since virtual treatment has been proposed as a way to maintain quality of care for patients due to a lack of other options; therefore, virtual treatment has become increasingly popular during the recent pandemic [5]. However, only a few doctors and patients are adequately trained about how to use these digital services efficiently [44]. As a result, guidelines and recommendations are needed regarding the usage of different signals (i.e., online signals or offline signals) to educate both physicians and patients on how to transmit different signals on virtual platforms in order to improve the patients’ online consultation choice [16,23,36]. Therefore, this study aims to present a unique problem in the COVID-19 pandemic scenario, which incorporates different types of signals in the same research, that is, online signals and offline signals transmitted by marketers and sellers to investigate their impact on patients’ physician selection choice for e-consultation using the theoretical foundation of signaling theory.

Signaling theory is useful in understanding the individuals’ attitudes and scale down the knowledge asymmetry [27]. The signaling theory provides a theoretical foundation for how one party uses signals to communicate secrete or restricted information to another party regarding the quality of product or service in order to enable the purchase or exchange [45]. Due to information asymmetry in online markets, it is very important to make full use of the signaling theory. In signaling theory, the cost of a signal is the main signaling component, which contributes to signal values [46]. Our research tends to answer the following first question to advance theory and research on the impact of different signals on patients’ decisions about physicians for online consultation during the COVID-19 pandemic.

(1) How do online and offline signals transmitted by marketers and sellers on e-consultation websites affect the patients’ physician selection choice for online consultation during the pandemic?

Second, the signaling environment plays a critical role in defining which signal to use [47], and the signal strength is mitigated by the signaling environment where the signal operates [48]. Researchers suggest the impact of signaling will be greater in case of high environmental uncertainty. For example, Siering, et al. [49] examined that the influence of signals on review helpfulness vanishes in case of low information environment uncertainty, whereas relationship prevails in a high information environment uncertainty. Similarly, Lester, et al. [50] reported that top management teams’ prestige is more valued for firms who are responsible for the initial public offering in the more uncertain environments than more certain environments. On the other side, several other scholars revealed that the influence of signaling would be weakened in case of high environmental uncertainty. For example, using a sample from U.S-based firms, Certo, et al. [51] explored the moderating role of uncertainty in the relationship between top management teams and global strategic posture. In virtual treatment, the patient decision-making for online consultation also varies across different environments (disease risk) [52]. Hence, this study develops a new theoretical model and tests the argument for patients’ physician selection choice for online consultation across varying environments (i.e., disease risk) during the pandemic. This study proposes our second research question is provided as follows:

(2) Does the patients’ perceived disease risk have moderating effects on the relationships between both online signals and offline signals, and patients’ physician selection choice for e-consultation during the pandemic?

As it is important for patients to search online for the best physician or review their current one, this work offers an overview of how patients make choices online using a real longitudinal panel dataset collected during the recent coronavirus outbreak in the U.S., which was not done in earlier digital healthcare studies. We also hope that the findings of this study would assist policymakers and providers in developing effective and efficient intervention strategies.

The remaining part of this study is organized as follows: Section 2 contains the research hypotheses, Section 3 explains the methods employed, Section 4 features data analysis and results, Section 5 discusses the major findings and contributions, details the implications as well as research the limitations and future research directions. Finally, Section 6 presents the conclusion of this paper.

## 2. Research Hypotheses

**Figure 1:**
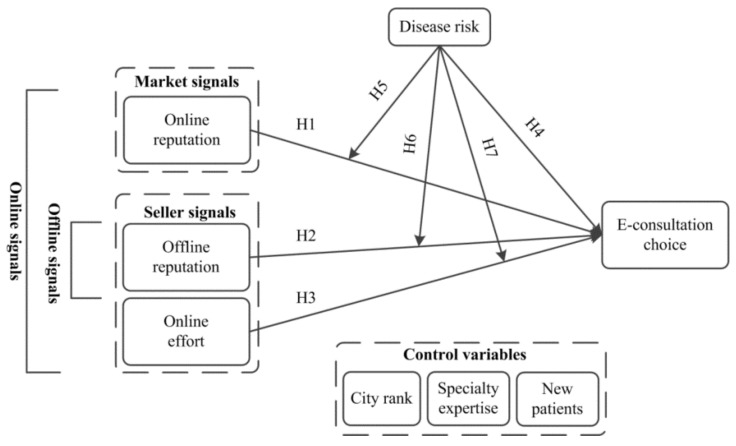
Research model.

Based on the earlier evidence, this paper links online reputation and online effort as online signals generated by market and seller. In contrast, we use offline reputation as offline signals to describe seller signals only. The study also proposes that environmental uncertainty directly affects patients’ physician selection choice for e-consultation (e-consultation choice from here onward). That also has moderating effects on the relationships between online signals, offline signals and patients’ e-consultation choice. The proposed model is shown in Figure 1.

### 2.1. Online Reputation and Patients’ E-Consultation Choice

Reputation cues presenting others’ influence are also important in consumer purchase decision-making. In online markets, consumers need to judge the seller’s ability to complete the purchase process successfully. Consumers evaluate the online information posted by others, which is a challenging task because participants can belong to any geographical location on the Internet [53]. They tend to perceive online reputation information as useful in influencing their judgments regarding products or services. Consumers have access to limited information about a seller or provider searching for a quality product and potential seller. When consumers do not know each other and do not have prior interaction, online reputation reflects the only information about the seller or provider quality that does not originate from the seller or provider themselves [54]. The market signal, that is, online reputation, refers to information regarding different sellers’ products and services created by buyers based on their consumption experience. Information is passed passively or unintentionally between the various actors in a market [55]. Researchers claimed that product or service consumers who are satisfied or dissatisfied with a brand would share their experience through market signals [56].

Online reputation phenomenon can also be extended to the delivery of online healthcare services. The physician’s *online reputation* refers to patients’ perceptions regarding the physician’s assessment online after each interaction [33]. To select a competent physician, healthcare consumers take suggestions from their family members and friends to obtain online WOM information. A number of investigations have debated the impact of physician online reputation, such as the star ratings and textual feedback on patients’ choice of physician selection online [16,32,35,43,57,58]. The existing literature validates online WOM as a valuable and efficient channel for disseminating information regarding provider reputation to consumers.

On e-consultation websites, patients have more cues to determine the quality of the online services offered by their physicians [59]. When the online market has so many physicians, consumers can only select those with higher star ratings. Contrary, if the market entirely contains physicians with poor reputations, users will not consider these poorly rated physicians for online consultation [60]. Thus, a market with a strong reputation and highly rated physicians is very critical. Suppose the market contains too many physicians with a high online reputation. In that case, market productivity will be enhanced because of a larger consideration set of credible doctors from which users choose physicians (i.e., the demand for highly reputed and quality physicians is increased). In the same line of thought, market productivity will not be enhanced if the market contains limited, highly rated physicians [10]. Extant literature suggests that the consumers’ attitudes toward online health services in the form of ratings affect their purchase intentions [40,41]. According to recent studies, a physician’s online reputation is a good indicator to reflect the patients’ online behavior [10,16]. Thus, we hypothesize the following:

**Hypothesis** **1** **(H1).** *Physician with a strong online reputation is positively associated with patients’ e-consultation choice.*

### 2.2. Offline Reputation and Patients’ E-Consultation Choice

Physician *offline reputation* is a patient belief about the ability and expertise of a doctor to provide effective and reliable services [61]. Consumers commonly search for reputable physicians before consulting a physician in the e-health context [32]. Previous literature suggests that physicians display their organizational status on their profile page regarding their titles and technical skills to deal with a particular disease [12,16,38,62]. Patients search offline information to seek technical and other physician features as the paramount criteria to evaluate the provider’s performance and choose a competent provider [37]. According to Guo, et al. [63], physician status capital (offline reputation) is positively related to physician online selection choice. A study by Li, et al. [64] indicated that professional capital is important in increasing service demands in the online healthcare market. Moreover, the physician capital leads to enhance performance outcome [38]. Professional capabilities are important in driving professional services to patients [62]. Physicians with higher levels of expertise tend to bring novelty to their practices to attract more patients [37]. Several other studies also discussed the relationship between physician offline reputation and patients’ online choice [16,33]. These studies confirmed the healthcare consumers’ perceptions that physician offline reputation is the key to their online decision-making. Physicians with a higher clinical tag and/or academic title signal patients that the physician is formally recognized as reputable. We thus hypothesize that:

**Hypothesis** **2** **(H2).** *Physician with a strong offline reputation is positively associated with patients’ e-consultation choice.*

The term ‘*effort*’ can be defined as the amount of energy consumed on an act per unit of time, while the length of time spent working and the intensities of the work activities are two important aspects of effort [65]. Several researchers have considered the impact of effort on consumer purchase decisions [66,67].

Due to the intangible nature of services, employees’ verbal and nonverbal acts significantly impact the consumers’ attitudes toward the quality of service [68]. An employee’s effort is more critical in service settings, as customer service quality evaluations are often directly associated with service provider performance [69]. If the employee is deemed to be paying extra attention to their job, then s/he may get a higher rating from its customers [70]. Efforts by the employee may impact consumer purchase intention, which is vital to the overall success of a service organization. The positive impact of the effort will lead to the probability that the customer will shop and buy products in the future. Since online healthcare services belong to the service domain, physicians’ efforts related to the services they deliver may influence the patients’ perceptions regarding service quality and may alter their decisions and opinions about physicians. The study by Deng, Hong, Zhang, Evans and Chen [36] indicated that patients prefer online consultation with physicians who have higher quantities of effort online. Liang, Luo and WU [65] described a positive relationship between a physician’s online effort and patients’ choice for online consultation. Physicians’ with more online contributions have a significant positive impact on their benefits [40]. Furthermore, the findings from Li, Tang, Yen David and Liu [16] revealed that physician online effort in the form of self-representation is positively related to the patient online selection of a particular physician. Apart from these studies, the findings from several other kinds of research also revealed a significant positive effect of physician online efforts on patients’ choice to choose a good doctor online [58,71].

While choosing doctors for online consultation, patients can visit the doctors’ profile page and get further information, such as personal blogs, articles written and prior correspondence between healthcare providers and patients. Through this point, patients gain a sense of the physician’s efforts in the past, which can influence their attitudes toward the physician and, therefore, increases the probability of selecting a good physician. More effort displayed online by the physicians towards their services offering could increase the probability of patients selecting them for online consultation. Thus, the following hypothesis is proposed:

**Hypothesis** **3** **(H3).** *Physician with a higher amount of effort online is positively associated with patients’ e-consultation choice.*

### 2.3. Direct and Moderating Impact of Disease Risk

Disease risk measures the severity of the effects of a disease type. According to the signaling theory in the e-health context, the signaling environment significantly affects patient satisfaction levels with service quality [57,72]. In this research, we tend to differentiate the risk between different types of diseases rather than the differences in risk between different patients. This is basically because the former is supposed to be consistent among various individuals. Since mortality is a key indicator to judge disease risk [32], the notion of disease risk has been discussed previously in medical science research [16,32,35,43,57].

A physician treating high-risk disease can deliver patients more opportunities to evaluate his/her technical skills. Online physician reviews are open content that is visible to the public on virtual platforms. They provide patients with some overview regarding the physician skills, including clinical quality and bedside manners. Suppose reviews are posted by patients who involve in high-risk disease and visited their concerned physicians. In that case, these physicians can provide potential patients with high-quality care to enhance patients’ online behavior. For example, patients with a severe illness (i.e., cancer) experience more pain and discomfort than patients with a mild illness (i.e., influenza) [57]. The probable explanation for this is mainly because mortality is a significant predictor for assessing the disease risk, and cancer disease has a higher mortality rate for patients than influenza. Therefore, patients involved in high-risk disease need more competent physicians than those involved in low-risk disease. Hence, we propose:

**Hypothesis** **4** **(H4).** *A physician who treats high-risk disease has more chances to influence patients’ e-consultation choice in comparison to those who treat low-risk disease.*

According to the signaling theory, differences in the disease risk level affect the patient perception of the provider’s online reputation regarding service quality and satisfaction. Scholars have found that the higher the online reputation level of a physician for high-risk disease, the more his/her service quality and service level acknowledged by the sizeable number of patients who consult online [16]. Patients with high-risk disease bear greater financial and health damages; therefore, they need more assurances of high-quality service from a reputed physician than patients with low-risk disease [39]. For instance, Shah, Yan, Shah, Shah and Mamirkulova [32] indicated that the highly-risk disease strengthens the effect of physician online reputation on a patient decision-making process. For high-risk disease, a physician sends high online reputation signals to help patients choose competent physicians, thus effectively assisting patients in distinguishing skilled physicians [40]. Therefore, in such situation a physician higher online reputation can communicate a signal of competency and quality of services for high-risk disease.

Online reputation is more unbiased and reliable than conventional information from peers [73]. Patients with high-risk disease seek a physician’s online reputation (e.g., satisfaction ratings and patients’ feedback comments) to evaluate the physician’s quality of care [43]. Moreover, patients may find more knowledge about physicians’ online reputation to measure their service delivery process (e.g., physicians’ and office/staff performance), which is critical in affecting patients’ online consultation decisions. Several other studies have also reported the moderating effect of disease risk on patients’ choice [35,39]. In addition, since the disease risk is a critical factor in patients’ decision-making, we assume patients with high-risk diseases will strengthen this motivation between online reputation and patients’ e-consultation choice. Therefore, based on the above discussion, we propose:

**Hypothesis** **5** **(H5).** *Disease risk moderates the effect of a physician online reputation on patients’ e-consultation choice. High-risk disease exhibits a stronger positive connection between the online reputation and e-consultation choice compared to low-risk disease.*

Reputation reflects as a signal of quality. Previous investigations of the signaling theory described that the signaling environment plays a key role in defining the usage of signals [16], and the signaling environment moderated the signal strength [49]. The disease risk in the virtual platforms relates to the purchase significance of healthcare services for patients. In the case of low-risk disease, any physician can treat his/her patients with a high possibility of better care. Therefore, the strength of the relationship between a physician quality (offline reputation) and treatment outcome likely to be low. Conversely, in the case of high-risk disease, the probability that any physician can treat the patient becomes low, and in this situation, the treatment outcome largely depends on the physician’s quality (offline reputation). The association between physician quality (offline reputation) and treatment outcome will be strong enough in this situation. Moreover, high-risk diseases are commonly linked with unknown circumstances such as sudden deaths, higher treatment cost burden and lengthy hospital stays. Therefore, patients suffering from high-risk disease tend to be more sensitive to the physical status as an offline reputation than those suffering from a low-risk disease. Hence, we hypothesize:

**Hypothesis** **6** **(H6).** *Disease risk moderates the effect of a physician offline reputation on patients’ e-consultation choice. High-risk disease exhibits a stronger positive connection between the offline reputation and e-consultation choice compared to low-risk disease.*

The disease risk may also moderate the relationship between online effort and patients’ e-consultation choice. Physicians treating high-risk diseases require a high level of online expertise. Therefore, they can put more effort into online knowledge sharing on e-consultation platforms. With such expertise and skills, doctors will be more willing to exchange free health information online through their personal desires and internal ambitions (social motivation), increasing their financial results by attracting more patients [71]. However, those treating low-risk diseases may put little online effort due to less information-sharing experience on platforms. As a result, relying on their professional motivation to encourage more efforts to share health information is weaker for physicians treating low-risk diseases than physicians treating high-risk diseases [16]. Based on the above arguments, when the disease risk is low, the effect of online effort on patients’ e-consultation choice will be low because it does not significantly reduce uncertainty. In contrast, when the disease risk is high, the effect of online efforts on patients’ e-consultation choice should be high since it significantly reduces the associated uncertainty. Hence, we propose that:

**Hypothesis** **7** **(H7).** *Disease risk moderates the effect of a physician online effort on patients’ e-consultation choice. High-risk disease exhibits a stronger positive connection between the online effort and e-consultation choice compared to low-risk disease.*

## 3. Methods

### 3.1. Research Contexts

Our research contexts are two leading commercial online consultation platforms in the U.S, Healthgrades.com and Vitals.com. We construct a novel longitudinal data set using several data sources to test our hypotheses. According to the global internet engagement statistics from Alexa.com, Healthgrades and Vitals had a traffic rank of 7502 and 31,388, respectively. As of June 2020, on average, a visitor spends 1:49 and 1:44 in minutes and seconds on each site every day, respectively. In addition, more than 8 million physicians enrolled in these platforms as of June 2020. We checked both these platforms provide similar structure and services. Each physician on both these sites can create a personal profile page. Both these channels display their basic information, which includes: their demographic statistics, professional title, education, experience and area of expertise, board certifications, contributed articles and service records, etc. In addition to this information, a physician can receive feedback from patients on these platforms in the form of ratings as well as text-based reviews.

### 3.2. Data

A network spider was developed coded in python 3.6 to scrape data from the homepages of 6344 physicians from both these virtual platforms. Data were collected on 30 January, 30 March and 30 May 2020, during epidemic COVID-19, which covers the period from December 2019–May 2020 from the top 25 metropolitan areas in the U.S. We chose these geographical locations based on the highest Internet usage and the number of physicians with active board licenses in the U.S. [74,75]. This scheme of data collection allows us to create a longitudinal panel dataset with a 60-day interval between each time period. The URLs and home page of physicians were matched with 3 different time periods. This process revealed that 1420 physicians’ data were not available across all 3 periods (in particular, 722 physicians’ data were not available in 2 periods and 698 had 1 period missing), there were more than 1 null value across 3 periods for 496 physicians, and 197 physicians having some abnormal values. After removing the missing and abnormal values, our final dataset includes the 4231 physicians’ information. Each individual physician selection was non-random in order to avoid including the same provider more than once.

Due to access, time and cost restraints, we restrict the data collection from virtual platforms to the 14 different diseases. Following the disease mortality rate from the Centre for Disease Control and Prevention [76], these 14 diseases cover acute and chronic diseases, as well as both high mortality rate (heart disease, cancer, unintentional injuries, chronic lower respiratory diseases, cerebrovascular diseases, Alzheimer disease and diabetes mellitus) and low mortality rate diseases (influenza and pneumonia, kidney disease, suicide, chronic liver disease and cirrhosis, septicaemia, hypertension and parkinson disease).

### 3.3. Variables Measures

#### 3.3.1. Dependent Variable

*E-consultation choice*: Patients’ e-consultation choice is measured by two dimensions. Patients’ quantity, who had received online consultations in the last 6-months interval, and the ratio of satisfied patients to all patients. Both these dimensions were averaged to obtain a composite variable.

#### 3.3.2. Independent Variables

*Online reputation*: Online reputation is measured by two dimensions. Review volume (i.e., the number of reviews for that physician) and rating score (on a scale of 1–5, with 1 being the lowest status and 5 being the highest).

*Offline reputation*: Offline reputation is measured by four dimensions. Physician professional title in the hospital, education, experience and board certifications. We thus calculate the mean value of four dimensions as a measurement of physician offline reputation.

*Online effort*: The online effort is measured by the number of blogs that the physician has initiated, the number of physician’s health care articles, physician number of replies to patients, and the length of physician reply. Thus, the physician responsiveness component of online efforts could be calculated by the average text length of replies to each review, see Formula (1), where length (*rp_ij_*) is the text length of replies to the *j_th_* review *r_ij_* by physician *i*, and *n* is the total number of reviews on the physician *i* profile page.

(1) $$Online\;Effort_{i}=\frac{1}{n}\sum_{j=1}^{n}length(rp_{ij})$$

#### 3.3.3. Moderator

*Disease risk (Risk)*: The disease risk is measured by the severity of the physical and physiological effects. We used the following strategies for classifying the 14 diseases into different risk levels. If the illness is mortally severe, it is considered a high-risk disease. In contrast, low-mortally illness is categorized as a low-risk disease.

*Control variables*: Control variables in this study include *city rank*–whether physician belongs to first, second or third-tier city according to Internet usage and board-certified physicians, *specialty expertise*–the number of diseases that a physician treats, and *new patients*–whether physician accepts new online appointment–bookings or not. Variables and their description are listed in detail in Table 1. A log transformation was used for quantity, volume, experience, blogs, articles, length and phy_exp because its numerical value was much greater than other values. In some distributions, the log-transform reduces skewness, particularly when large outliers are present.

#### 3.3.4. Estimation Model

Our empirical econometric model fit the e-consultation choice, online reputation, offline reputation, online effort, disease risk, city rank, specialty expertise and new patients’ data to the following log-linear relationship. The dependent variables and independent continuous variables have been converted into a log form, as the distribution may not be normal.

(2) $$\begin{array}{l} \log(E-consultation\;choice_{i})=\beta_{0}+\beta_{1}Rank_{i}+\beta_{2}\log(Phy\_Exp_{i})+\beta_{3}New_{i}+\\ \beta_{4}\log(Online\;reputation_{i})+\beta_{5}Offline\;reputation_{i}+\beta_{6}Online\;effort_{i}+\\ \beta_{7}Risk_{i}+\beta_{8}\log(Online\;reputation_{i})\times Risk_{i}+\beta_{9}Offline\;reputation_{i}\times Risk_{i}+\\ \beta_{10}\log(Online\;effort_{i})\times Risk_{i}+\varepsilon_{i}\\ \mathrm{where}\;i=1,2,\ldots \ldots ,n\;\mathrm{index}\;\mathrm{all}\;\mathrm{physicians}\\ \beta_{0}-\beta_{10}\;\mathrm{are}\;\mathrm{the}\;\mathrm{parameters}\;\mathrm{to}\;\mathrm{be}\;\mathrm{estimated}\\ \varepsilon_{i}\;\mathrm{is}\;\mathrm{the}\;\mathrm{error}\;\mathrm{term} \end{array}$$

### 3.4. Data Analysis Procedure

All our empirical analyses were run using STATA 12.0 (StataCorp, College Station, TX, USA). We used linear regression for time-series data. To validate the interaction effects results, we followed the procedures from Aiken and West [77]. Furthermore, a different method was used to check the robustness of e-consultation choice.

## 4. Results

Descriptive statistics for studied variables are listed in Table 1. Pearson correlation for the key variables used in this study is shown in Table 2. Results show that the values of variance inflation factor (VIF) statistics for every independent variable were below the threshold (i.e., 10); hence multicollinearity is not a serious issue in this study, and results are reliable.

### 4.1. Hypotheses Testing Results

The regression results are presented in Table 3. Equations are shown in a hierarchical order. The effects are first shown with only control variables in Model 1, Model 2 to test direct effects and Model 3 tests interaction effects. The adjusted R^2^ and F-value both represent a good fit.

For Model 1, it is observed that both physician experience (*β* = 0.033, *p* < 0.05) and new patients (*β* = 0.010, *p* < 0.01) have positive impacts onto the patients’ e-consultation choice, whereas city rank (*β* = −0.016, *p* < 0.01) has a negative influence onto the patients’ e-consultation choice.

In model 2, two kinds of signals are explored in this study, such as physician’s online reputation as market signals, whereas physician’s offline reputation and online effort as seller signals. The two signals, namely, physician’s online reputation (*β* = 1.132, *p* < 0.05) and online effort (*β* = 0.069, *p* < 0.001), are positively related to patients’ e-consultation choice, thus hypotheses H1 and H3 are supported. The relationship between physician’s offline reputation and patients’ e-consultation choice is insignificant; the hypothesis H2 could not be confirmed. In addition, the disease risk positively influences the patients’ e-consultation choice (*β* = 0.243, *p* < 0.05), thus hypothesis H4 is supported.

Model 3 analyzed the moderating role of disease risk. The interaction term (disease risk) between both physician’s offline reputation and patients’ e-consultation choice (*β* = 0.026, *p* > 0.05), and physician online effort (*β* = 0.030, *p* > 0.05) and patients’ e-consultation choice are not significant; thus, H6 and H7 are not supported. In contrast, the interaction term between physician’s online reputation and patients’ e-consultation choice (*β* = 0.128, *p* < 0.05) is positive and significant. This means H5 is supported.

**Figure 2:**
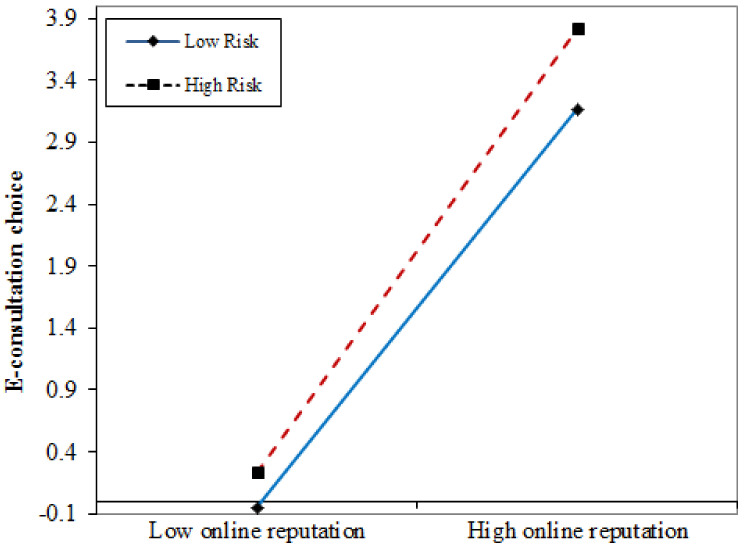
Interaction effect.

The interaction effect in Figure 2 shows that physicians with a higher online reputation (in dashed red line) have a larger slope than those with a lower online reputation (in solid blue line), indicating the positive interaction between physician’s online reputation and patients’ e-consultation choice. At high-risk disease (in dashed red line), online effort increases more rapidly than at low-risk disease (in solid blue line), indicating that high-risk disease increases the positive effect of physician’s online effort on patients’ e-consultation choice. This figure provides an extra indication to support H5.

### 4.2. Robustness Check

To check the robustness of patients’ e-consultation choice, we collected a new dataset when the number of new infection cases and mortality rates due to COVID-19 was at its peak (16 June–30 June 2020) [78]. Our empirical analysis was run on this new dataset covering physician homepage information for 15 days’ time period. The robust empirical results in Table 4 are consistent with the main results in Table 3.

## 5. Discussion

The COVID-19 pandemic has triggered public health concerns around the globe. In certain special cases (e.g., the COVID-19 crisis), e-consultation could accomplish the target of public isolation and fulfill the patients’ needs for high-quality care [79]. In comparison to physical consultation, e-consultation can only be used to diagnose mild and high-risk diseases in the absence of appropriate medical exams and treatment systems. However, written recommendations from physicians are still useful to the general public [80]. While e-consultation cannot wholly replace physical consultations, it can be used as a first step after being incorporated into a physical consultation [30]. As a result, while enhancing the functionality of various signaling mechanisms, providers should also improve the patients’ choice of physician selection on e-consultation websites [16]. Our findings show that promoting the role of e-consultation is critical to attracting the public to use it, as positive online signals suggest that the public has more confidence in doctors’ capacity and honesty.

The main objective of the current study was to develop a theoretical model and examine how different online and offline signals generated by market and sellers predict patients’ online consultation choice. The model was tested using 3 points longitudinal panel data obtained from Healthgrades and Vitals websites during the pandemic period in the U.S. As a result, this study presents a number of key findings, theoretical contributions and implications for practitioners.

### 5.1. Key Findings

The current study presents four major contributions.

First, the positive online reputation significantly and positively influences the patients’ physician selection choice for e-consultation. According to the signaling theory, when there are positive online WOM signals about a physician’s reputation, new patients are more likely to consult him/her online [16,32]. During the pandemic, patients can enjoy the quality of life and higher-quality online services from a physician with a high online reputation.

Second, the current study result shows the insignificant relationship between offline reputation and patients’ e-consultation choice. This finding is different from previous studies, which generally showed the significant and positive effects of the offline reputation [16,26,37]. A possible justification of this result could be travel bans, lockdowns and the possibility of infection spreading during the COVID-19 pandemic; patients rely more on online WOM from peers to seek physician details rather than search for offline information. However, our study findings are supported by a couple of previous studies in which public voice and WOM positively affect social presence [81,82]. Our findings showed patients would prefer those physicians for online consultation, with a high online social presence relatively high reputation in offline hospitals. Results suggest that physicians can work hard online to improve their reputation and build a strong tie with their patients.

Third, the online effort is positively related to patients’ e-consultation choice. This finding indicates that physicians willing to spend more time and effort online to benefit patients will eventually get more patients [18,36]. Even though many physicians are registered on virtual platforms to provide online consultation services to their patients during the pandemic, the public is always keen to try those physicians who put more effort online. Therefore, it is essential to strengthening the publicity of e-consultation for the public. In the promotion of physicians on e-consultation platforms, e-consultation providers should offer advanced services for innovative and stable services for the common [79].

Fourth, disease risk is positively related to patients’ e-consultation choice. As mentioned earlier, regarding the current situation of the epidemic, travel restrictions and quarantine rules, when the risk of disease is high, patients are more likely to seek health information from online platforms rather than offline hospitals. As a result, virtual health platforms will still be the first choice for patients to consult physicians for high-risk disease treatment.

Fifth, the impact of online reputation on patients’ e-consultation choice is higher for high-risk disease than for low-risk disease. When the risk of disease is low, the treatment outcome is certainly low in uncertainty, and thus the signalling is less effective. In contrast, when the risk of disease is high, the treatment outcome is high in terms of uncertainty. The key explanation for this argument is that the patient is highly dependent on the physician’s quality of online medical services (online reputation). This makes the signalling more efficient for e-consultation [16].

Sixth, according to our findings, the impacts of offline reputation and online effort are not influenced by the disease risk, which is consistent with the findings of previous studies. For example, Li, Tang, Yen David and Liu [16] reported that offline reputation and online effort both differ from online reputation with regard to signalling source. The offline reputation and online effort are the main signals on the e-consultation platform transmitted by the sellers (i.e., seller signals), whereas the service popularity is the signal initiated by the buyers (i.e., market signals). The study results reveal that the disease risk moderates the impacts of market signals but does not affect seller signals. This is due to the fact that market signals are the direct input from previous patients on the quality of online services. In contrast, seller signals are usually the information that is effectively transmitted to patients by the physicians. Patients have to judge the quality of online services regarding offline reputation and online effort sent by the physicians themselves. Notably, in terms of service efficiency, market signals are more efficient than seller signals. Hence, the influence of market signals on e-consultation platforms becomes more critical when the risk of disease becomes high, and the effects of seller signals do not rely on the level of disease risk.

### 5.2. Theoretical Implications

This research also provides several theoretical contributions to the existing research.

First, we utilize the information asymmetry theory to frame our hypotheses in the online health services context. Accordingly, we systematically analyzed and incorporated all the individual signals in single research (i.e., online and offline signals), which affect patients’ consultation choice in the online healthcare market. In particular, we considered online reputation and online effort as online signals, whereas offline reputation as offline signals. Even though previous studies have explored the impact of two or more signals in online healthcare market [18,33,36,37,63]; however, all these market and seller generated signals were not scientifically investigated in a single study, and hence, we cannot compare their effects explicitly because of the different laboratory conditions and independent study outcomes. Thus, our results may improve the treatment outcome for individuals by providing accurate information about the healthcare provider during COVID-19.

Second, this study extends the research on the concept of environmental uncertainty in understanding patients’ physician selection choice for e-consultation. Referring to the literature in strategic management, the concept of environmental uncertainty is claimed as the disease risk in this research. Therefore, this research explored the moderating impact of disease risk on different signaling mechanisms on e-consultation websites (online reputation, offline reputation and online effort, etc.), which, according to our information, were not tested in earlier studies. The outcome of this analysis reveals the moderating impact of disease risk onto the online reputation signals on the e-consultation platforms, suggesting that there is a higher effect of online reputation for high-risk disease. This result means the signals are not uniformly useful across all disease types. The online reputation signals would be ideal and best-suited for high-risk disease.

Third, by incorporating variables related to healthcare into the research model, this research also provides evidence for information systems and healthcare researchers. Even though online reputation, offline reputation and online effort signals were debated in previous online healthcare signaling literature; however, their differences were rarely investigated. This study empirically examined that online reputation signals should be treated differently from both offline reputation signals and online effort signals. In fact, online reputation is the market signal transmitted by the buyers in the online market. In contrast, offline reputation and online effort relate to the seller signals transmitted by the healthcare providers themselves. This work, therefore, adds to previous literature on signaling and related fields by demonstrating that market signals may be more predictive than seller signals. Hence these signals tend to be more influential when there is a high risk of disease. As a result, it is important to adjust the policies to adapt to various disease categories.

Fourth, methodologically this study contributes to the literature [12,36] by using a large longitudinal panel dataset in three different time periods that combines intelligent signaling mechanism, such as online and offline signals; to examine how the combination of different signals effect patients’ e-consultation choice during the COVID-19 pandemic. To understand patients’ behavior, the current study collected real information from physician home pages during the pandemic, which, according to our knowledge, was not investigated in earlier studies.

### 5.3. Practical Implications

Our study has some substantial practical implications.

For physicians, this study indicates that they may benefit from using e-consultation platforms; in the current pandemic crisis, they should be motivated to use these platforms continuously. Our results reveal differences between e-consultation platforms and conventional e-commerce websites. From a practical point of view, an e-commerce website is a forum for supportive product or service information, with feedback mechanisms provided by certain websites. However, maintaining the strong ties between providers (doctors) and buyers (patients) are more important than conveying information online. Particularly, regarding the knowledge sharing as an online effort by physicians on e-consultation platforms, this study highlights that by using virtual platforms, physicians can improve doctor-patient contact to enhance patients’ attitudes toward a provider during the COVID-19 pandemic situation. This evidence would be further strengthened for junior physicians with low status and investing more time and achieving a reputation for their career. This is also a significant factor in improving doctor communication skills, such as developing incentive structures to strengthen the doctor relationship with his/her current patients, which is more beneficial for physicians than recruiting new patients.

For platform developers and policymakers, this study shows that online reputation and online effort factors can affect patients’ physician selection choice for e-consultation. Thus, website managers can develop convincing windows on different e-consultation platforms and include all these necessary components to develop an effective e-consultation platform. As a result, efficient signals can be conveyed by these components. From a policy perspective, the current study suggests that e-consultation platforms be required to provide reliable data on doctor-patient interactions during COVID-19. Our findings also show that the signals are not globally reliable, with a stronger impact on high-risk disease.

To end with, the disease risk has a positive moderating role in the relationship between online reputation and patients’ e-consultation choice, an understanding of what quality and value mean to patients with different disease risk offers the promise of improving the physician’s brand position through precise market analysis and segmentation, service planning and pricing strategy. A physician that treats high-risk disease patients should devote more attention to online reputation building in order to improve patients’ attitudes towards him/her. For example, a physician could improve his/her online service attitude, improve response speed and provide detailed information on different aspects of healthcare.

### 5.4. Limitations and Future Research

Future efforts can be focused on several possible extensions of this work. First, we have only chosen a certain duration of the outbreak to evaluate patients’ online behavior. The pandemic is still at its peak in the U.S., with thousands of new infections being reported during and after the period of data collection, and patterns are changing on a daily basis. With several reports of successful vaccine trials in the U.S. and other countries, we might have missed a few insights from the results. As a scheme of our future research, the data collection will continue to test these variables when the number of new infection cases declines substantially in the U.S. Second, although both websites confirm that the physicians’ information on their homepages was actually posted by the physicians themselves the authenticity of this information cannot always be guarantee; future research could cross-validate the physician information using other websites such as https://www.certificationmatters.org, https://www.cms.gov/Physician-Compare-Initiative/ and https://www.jmr.fsmb.org/.

## 6. Conclusions

Analyzing patients’ online behavior using data collected during the novel coronavirus shed light on how the patients use multiple signals to consult a particular physician online. This study is the first step to understanding the impact of online and offline signals on patients’ physician selection choice for e-consultation during the COVID-19 crisis. Based on the signaling theory, physician online reputation and online effort were identified as antecedents of patients’ e-consultation choice. In addition, disease risk has a positive effect on patients’ e-consultation choice. Moreover, a positive effect was identified on the online reputation, demonstrating a higher effect for high-risk disease. These findings validate the claim during the recent pandemic crisis, patients are more motivated to search physician information from online channels rather than offline channels. Finally, several suggestions are provided for physicians, platform developers and policymakers to manage the necessary signaling components on e-consultation platforms.

## Figures and Tables

**Table 1:** Variables’ measurements.

Variable	Symbol, Description and Measurement	Mean	Std.	Min.	Max.
**Dependent variable**					
*E-consultation choice*					
Patient quantity	Quantity–number of patients consulted a physician online in the last 6-months (logarithmic value).	145.78	543.17	0	15,459
Patient satisfaction	Satisfied–the ratio of satisfied patients to all patients (Original data)	0.11	0.15	0.04	0.21
**Independent variable**					
*Online reputation*					
Review volume	Volume–number of reviews for a particular physician (Logarithmic value).	34.39	17.23	1	123
Rating score	Heat–voting heat for the physician, evaluated by patients (Ordinal)	4.41	0.55	1	4.59
*Offline reputation*					
Professional title	CTitle–physician clinical title (Dummy: 1–medical doctor/0–otherwise)	0.91	0.49	0	1
Elite education	Education–physician graduated from top-50 medical school (Dummy: 1–yes/0–no)	0.31	0.11	0	1
Tenure	Experience–physician experience (in years) (Logarithmic value)	16.89	5.31	1	51
Board certification	Certification–physician board certification (Dummy: 1–yes/0–no)	0.88	0.21	0	1
*Online effort*					
Blogs	Blogs–the number of blogs initiated by a physician (Logarithmic value)	3.31	21.2	0	27
Scientific articles	Articles–the number of articles published by a physician (Logarithmic value)	0.12	10.11	0	47
Replies	Replies–the number of replies to patients by physician	5.6	113.2	0	47
Reply length	Length–number of words in a reply (Logarithmic value)	16.45	9.21	39.21	98.72
**Moderator**					
*Disease risk*	Risk–measured as disease mortality (Dummy: 1–high-risk disease/0–otherwise)	0.77	0.123	0	1
**Control variables**					
*City rank*	Rank–city rank where the physician works. A scale of 1–3 is used, with 1 being the lowest and 3 the highest (Ordinal).	2.55	0.04	1	3
*Specialty expertise*	Phy_Exp–the number of diseases that the physician is good at curing (Logarithmic value).	52.41	31.12	1	13
*New patients*	New–whether the physician accepts new patients or not (Dummy: 1–yes/0–no).	0.87	0.12	0	1

**Table 2:** Variables’ correlations.

Variables	1	2	3	4	5	6	7	8
1. E-consultation choice	**1.00**							
2. Online reputation	0.45	**1.00**						
3. Offline reputation	0.141	0.062	**1.00**					
4. Online effort	0.51	0.58	0.55	**1.00**				
5. Risk	0.191	0.245	0.185	0.188	**1.00**			
6. Rank	0.32	0.28	0.32	0.02	0.12	**1.00**		
7. Phy_Exp	0.32	0.41	0.25	0.21	0.23	0.28	**1.00**	
8. New	0.12	0.14	0.21	0.23	0.20	0.29	0.32	**1.00**

**Table 3:** Regression results.

Variables	Model 1	Model 2	Model 3
Constant	0.242 *** (0.112)	0.267 *** (0.102)	0.287 *** (0.091)
Rank	−0.016 ** (0.011)	−0.041 *** (0.003)	−0.031 *** (0.002)
Phy_Exp	0.033 * (0.005)	0.014 * (0.001)	0.013 * (0.001)
New	0.010 ** (0.015)	0.016 ** (0.027)	0.025 ** (0.021)
Online reputation		1.132 * (0.006)	1.117 * (0.009)
Offline reputation		0.067 (0.015)	0.081 (0.019)
Online effort		0.069 *** (0.019)	0.071 *** (0.021)
Risk		0.243 * (0.041)	0.317 * (0.114)
Online reputation × Risk			0.128 * (0.059)
Offline reputation × Risk			0.026 (0.011)
Online effort × Risk			0.030 (0.021)
Adjusted-R^2^	0.208	0.217	0.230
Log-likelihood ratio	429.631	419.765	411.145
F	76.683 ***	7.174 ***	4.162 ***
N	4231	4231	4231

Note: Standard errors are in parentheses. * *p* < 0.05; ** *p* < 0.01; *** *p* < 0.001.

**Table 4:** Robustness results.

Variables	Model 4	Model 5	Model 6
Constant	0.215 *** (0.141)	0.232 *** (0.114)	0.272 *** (0.098)
Rank	−0.013 ** (0.015)	−0.036 *** (0.008)	−0.027 *** (0.005)
Phy_Exp	0.029 * (0.007)	0.012 * (0.004)	0.011 ** (0.004)
New	0.008 ** (0.019)	0.015 *** (0.034)	0.021 ** (0.026)
Online reputation		1.115 ** (0.014)	1.109 * (0.015)
Offline reputation		0.041 (0.025)	0.071 (0.027)
Online effort		0.057 *** (0.024)	0.065 *** (0.024)
Risk		0.219 * (0.059)	0.219 ** (0.137)
Online reputation × Risk			0.114 * (0.071)
Offline reputation × Risk			0.021 (0.017)
Online effort × Risk			0.026 (0.029)
Adjusted-R2	0.241	0.262	0.291
N	821	821	821

Note: Standard errors are in parentheses. * *p* < 0.05; ** *p* < 0.01; *** *p* < 0.001.

## Data Availability

Not Applicable.
